# Variability in Response to Hepatitis B Vaccine in Hemodialysis Patients

**DOI:** 10.14740/jocmr1999w

**Published:** 2015-03-01

**Authors:** Elie El-Charabaty, Chadi Saifan, Majed Mark Samarneh, Suzanne El-Sayegh

**Affiliations:** a Nephrology Division, Staten Island University Hospital, Staten Island, NY, USA

**Keywords:** Hepatitis B vaccine, Hemodialysis

## Abstract

**Background:**

Hemodialysis patients are exposed to blood and blood products more than the general population and are also at higher risk for hepatitis B (HB) contamination. For these reasons, it is highly recommended that this patient population gets the HB vaccine. The efficacy of the vaccine is measured by measuring titers of antibody in the serum of the patient. A minimum titer of 10 mIU/mL is considered to be a response. The conversion rate in hemodialysis patients ranges from 50% to 80%, as compared to the general population where the conversion rate is over 95%. As opposed to the general population, end-stage renal patients on hemodialysis do not always respond to the vaccine. The main objective in this study was to try to identify factors that may hinder the response. Correction of these factors in the future may help non-responders.

**Methods:**

This was a retrospective chart review at a single hemodialysis center to compare the laboratory and clinical differences between responders and non-responders. Inclusion criteria are hemodialysis patients who received the HB vaccine and patients with concomitant hepatitis C. Exclusion criteria are patients who refused the vaccine and patients who did not complete the vaccine course.

**Results:**

There are a total of 108 subjects included in the study, out of which 44 (42.3%) are responders to the HB vaccine. A multivariate logistic regression was performed using the statistically significant risk factors as identified by the univariate logistic regression, including age range, albumin, hemodialysis vintage, vascular access and diabetes status. The results from the multivariate logistic regression show that advanced age (P = 0.005) and diabetes status (P = 0.003) are found to be strong independent risk factors of responder status. The type of vascular access (AVF or other types) is also marginally statistically significant (P = 0.05).

**Conclusions:**

In this retrospective chart review comparing HB vaccine in responders versus non-responders, we found that advanced age and a history of diabetes are independent risk factors in predicting responder status.

## Introduction

Hemodialysis patients are exposed to blood and blood products more than the general population. They are also at higher risk for hepatitis B (HB) contamination [1]. Hepatitis and hepatocellular carcinoma can be fatal complications of hepatitis [2]. For these reasons, it is highly recommended that this patient population gets the HB vaccine. The vaccine, depending on the brand used, is administered as a series of either three or four injections. The efficacy of the vaccine is measured by measuring titers of antibody in the serum of the patient. A minimum titer of 10 mIU/mL is considered to be a response [3]. The conversion rate in hemodialysis patients ranges from 50% to 80%, as compared to the general population where the conversion rate is over 95% [4]. As opposed to the general population, end-stage renal patients on hemodialysis do not always respond to the vaccine [5]. Maintaining an immune response is also another problem with this patient population [6, 7]. There are several factors and hypotheses as to why hemodialysis patients do not respond. These patients usually have a significant degree of inflammation secondary to factors such as blood contact with the dialysis membrane and indwelling catheters. Malnutrition is another recognized reason for poor response [8]. These patients are also uremic and that also has a dampening effect on the immune system [9]. It has also been stipulated that iron therapy and anemia play a role in mounting a response [10]. The purpose of our study was to try to identify the factors that might hinder this response in hemodialysis patients. Identifying such factors may help us better optimize patients to help them achieve a response.

## Methods

Our study is a retrospective study, during which a total of 119 patients’ charts in a single hemodialysis center were reviewed. The inclusion criteria consisted of involving hemodialysis patients who completed the course of HB vaccination with or without concomitant hepatitis C infection. The anti-hepatitis B surface antigen antibody level was also mandatory to include the patient in the study. On the other hand, we excluded all patients who refused the HB vaccine and those who did not complete the course of the vaccination. The final cohort included 108 patients who fulfilled the inclusion and exclusion criteria. Data obtained included patients’ demographics such as sex, age range, weight, height and body mass index, hemodialysis vintage, the presence of an immunosuppressant condition such as HIV infection or immunosuppressive medications such as steroids. The dates of HB vaccine and type of vaccine were noted. The two types of vaccines that have been used are: recombivax HB 40 μg given at 0, 1, and 6 months, or engerix B 40 μg given at 0, 1, 2, and 6 months. Laboratory work has been recorded including hemoglobin and hematocrit level, liver profile, albumin level, calcium and phosphorus level, HbA1C level, pre-hemodialysis BUN, urea reduction ratio, kt/V, iron studies as well as parathyroid hormone level. Past medical history significant for diabetes mellitus, arterial hypertension, malignancy, thyroid disease, liver disease particularly hepatitis C, steroids use and immunosuppressive therapy have been included. The doses of erythropoiesis-stimulating agents dose as well as intravenous iron supplementation doses have been noted. Because of the anonymity of the patients studied, the non-invasive nature of the research and since the data collected are from the standard of care of each patient, the requirement for a written consent form was waived. This study did not involve any costs.

### Statistical analysis

The primary outcome variable is response to HB vaccination. A responder is defined as any patient who achieves a minimum of 10 mIU/L titers of HB surface antibody in the serum of the patient. The objective of the study was to determine whether response rate is associated with any of the following predictor variables: age, hemodialysis vintage, presence of an immunosuppressant condition or medication, parathyroid hormone level, albumin level, urea reduction ration, dialyzer type, vascular access type, ferritin level and diabetes mellitus. As a first step, a univariate analysis was conducted using logistic regression to examine the association of each of the predictor variables with the response status of the patient. Next, the variables that are found to be significant at 10% level of significance two-sided in the univariate analysis have been entered into a multivariate logistic regression model to identify independent risk factors.

Best subsets selection procedure has been used as a screening method to identify the best set of predictor variables for the final multivariate logistic regression model. For each independent risk factor, odds ratios with associated confidence intervals will be presented. Statistical tests based on results from the multivariate logistic analysis will be carried out at an alpha risk of 0.05 level, two-sided.

## Results

There are a total of 108 subjects included in the study, out of which 44 (42.3%) are responders to the HB vaccine as manifested by a minimum of 10 mIU/L titers of HB surface antibody in the serum. Responder status is unknown for four subjects.

The response rate decreases by age of the patient. Among the patients aged 18 - 55 years old, 76% responded to the vaccine whereas for the patients aged 56 - 75 years old and 76 - 95 years old, 36.5% and 22.2% responded respectively. The response rate was 40% in patients who were considered immunosuppressed whereas it was 43.5% in those who were not.

There were only five subjects with dialyzer type cellulose acetate 110 and all five of them did not respond. Seventy-seven patients (74%) had AVF as a vascular access out of which 48% responded. Among patients who used other types of vascular access, only 26.9% had responded.

**Figure 1:**
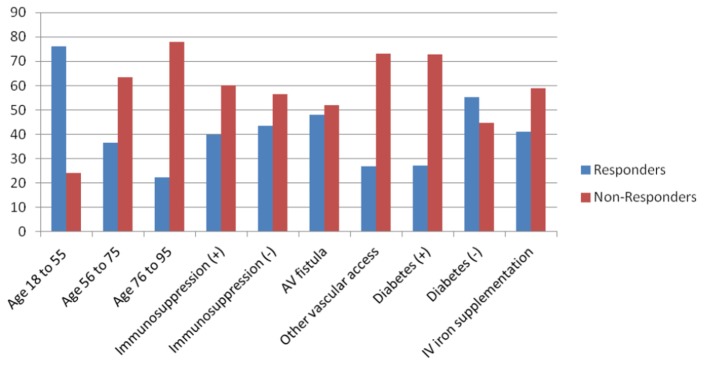
Different variables affecting response vs. non-response to hepatitis B vaccine.

Eighty-five of the patients (82.5%) had been given intravenous iron supplementation out of which 41.2% responded. In those who were not given intravenous iron supplementation, 50% responded. Forty-eight of the patients (46.2%) are diabetic out of which 27.1% responded and 72.9% did not respond. In the patients who are not diabetic, 55.4% responded. Those results are summarized in Figure 1.

The mean and standard deviation of hemodialysis vintage years were 6.0 ± 4.84 years and 4.2 ± 4.36 years for responders and non-responders respectively. The mean and standard deviation of parathyroid hormone level were 570.0 ± 639.7 pg/mL and 516.3 ± 617.2 pg/mL for responders and non-responders respectively. The mean and standard deviation of albumin were 3.9 ± 0.32 g/dL and 3.7 ± 0.31 g/dL for responders and non-responders respectively. The mean and standard deviation of urea reduction ratio were 70.3 ± 5.4 and 70.1 ± 7.66 for responders and non-responders respectively. The mean and standard deviation of ferritin were 576.9 ± 290.6 ng/mL and 654.6 ± 393.88 ng/mL for responders and non-responders respectively. Table 1 summarizes the above results for both responders and non-responders.

**Table 1:** Different Variables of Responders/Non-Responders

	Responders		Non-responders	
	Mean	Standard deviation	Mean	Standard deviation
Hemodialysis vintage (years)	6	4.84	4.2	4.36
Parathyroid hormone (pg/mL)	570	639.7	516.3	617.2
Albumin (g/dL)	3.9	0.32	3.7	0.31
Urea reduction ratio	70.3	5.4	70.1	7.66
Ferritin (ng/mL)	576.9	290.6	654.6	393.88

A multivariate logistic regression was performed using the statistically significant risk factors as identified by the univariate logistic regression. The results from the multivariate logistic regression show that age (P = 0.005) and diabetic or not (P = 0.003) are found to be strong independent risk factors of responder status. The type of vascular access (AVF or other types) is also marginally statistically significant (P = 0.05).

The odds ratios for the age range were 10.5 with 95% confidence interval of 1.67 - 66.09 for the range 18 - 55 years old, 6.56 with 95% confidence interval of 1.89 - 22.88 for the range 56 - 75 years old and 1.75 with 95% confidence interval of 0.61 - 5.06 for the range 76 - 95 years old. Whereas the odd ratio for the diabetes status was 3.15 with a 95% confidence interval of 1.43 - 6.94.

## Discussion

Hemodialysis patients are at high risk of contracting HB infection as well as other types of blood-related infections due to the fact that they are exposed to blood and blood products more frequently when compared to the general population [1]. Fatal complications can occur from chronic HB infection which includes reactivation of the HB virus which accounts for a clinical picture of acute hepatitis, superinfection by hepatitis D virus and cirrhosis that can degenerate into hepatocellular carcinoma [2]. Therefore, vaccinating hemodialysis patients against HB has become standard of care. However, it is well known that patients on hemodialysis are immunocompromised and this state is mainly due to over production of interleukine 6 and TNF alpha and relatively low production of interleukine 10 [11]. This immunosuppression status is responsible for a poor response of hemodialysis patients to HB as opposed to the general population [4]. The efficacy of the vaccine is measured by measuring titers of anti-hepatitis B surface antibody in the serum with minimum titer of 10 mIU/mL considered to be a response [3]. The conversion rate in hemodialysis patients ranges from 50% to 80%, as compared to the general population where the conversion rate is over 95% [4]. In our study, the response rate to the vaccine was 42.3% which correlates with the numbers found in the medical literature. Responders to the vaccine as per Grindt et al [11] tend to have a relatively higher production of interleukine 10 which suppresses the effects of the overly expressed interleukine 6 and TNF alpha when compared to non-responders. Also, maintaining an immune response is also another problem with this patient population [5, 6]. Factors that may hinder this type of response have been studied in multiple hemodialysis centers around the world and the medical literature shows that younger people on hemodialysis are more likely to be responders to the vaccine and maintain that type of immune response [12]. This was also demonstrated in our study which showed that the younger the age group is the more likely the response to the vaccination will be positive with the highest odd ratio being for the age group 18 - 55 years old. The meta-analysis done by Fabrizi et al also supports this result [13]. A possible explanation could be that younger patients do not have associated comorbidities which may interfere with their immune system and the degree of inflammation associated with blood contact with dialysis membranes and indwelling catheters is less accentuated than older patients [5-7]. This raises the question whether the hemodialysis vintage is associated with a poorer response to the vaccine. All the studies in the medical literature failed to demonstrate an association between the response to the vaccine and the hemodialysis vintage; in our study the P-value for the hemodialysis vintage was 0.069 with a rish alpha of 10% but that was not statistically significant in the multivariate analysis [14]. Malnutrition is another recognized reason for poor response [8]. In fact, our study has demonstrated that albumin level was significantly associated with the responder and non-responder status on the univariate analysis but did not demonstrate that it is an independent risk factor since the association was not statistically significant in the multivariate analysis. It has also been shown that iron therapy and anemia play a role in mounting a response [10]; however, we could not show that there is an association between the anemia, iron supplementation or even erythropoeis-stimulating agents and the outcome of the vaccination. The urea reduction ratio and other factors indicating the efficacy of hemodialysis particularly kt/V has been shown to be associated with a good response to the vaccine [3]; our study failed to demonstrate this association. The diagnosis of diabetes mellitus in patient on hemodialysis is an independent risk factor of being non-responder to the vaccine in our study as shown in the multivariate analysis with an odd ratio of 3.15. Elwell et al [15] have shown that diabetic patients are 2.5 times more likely to respond to the vaccine. There is a discrepancy in the results between Elwell study and our study, thus additional studies and particularly prospective studies will be needed to clarify this association.

Our study has few limitations. First, it is a retrospective study therefore a prospective study will be needed to affirm the association of the statistically significant results of our study and particularly clarify the relation of diabetes mellitus to the response outcome. Moreover, it will be interesting to assess the response rate to the new HB vaccine Heplisav which is thought to be more potent than the current vaccines on the market.

### Conclusions

Identifying factors associated with the response of hemodialysis patients to the HB vaccine will allow early intervention to increase the response rate, particularly vaccinating those patients at a younger age and before development of diabetes will help achieve a better immune response and therefore higher titers.
